# Bioinformatics analysis and validation of the critical genes associated with adamantinomatous craniopharyngioma

**DOI:** 10.3389/fonc.2022.1007236

**Published:** 2022-10-27

**Authors:** Chao Fang, Lin Zhou, Hui Huang, Hai Tong Xu, Tao Hong, Su Yue Zheng

**Affiliations:** ^1^ Department of Neurosurgery, Shenzhen Key Laboratory of Neurosurgery, the First Affiliated Hospital of Shenzhen University, Shenzhen Second People’s Hospital, Shenzhen, China; ^2^ Department of Neurosurgery, Shanghai Tenth People’s Hospital, Tongji University School of Medicine, Shanghai, China; ^3^ Department of Neurosurgery, The Second Affiliated Hospital of Nanchang University, Nanchang, China; ^4^ Department of Neurosurgery, The First Affiliated Hospital of Nanchang University, Nanchang, China

**Keywords:** craniopharyngioma, pathways in cancer, *CDH1*, *SHH*, *WNT5A*

## Abstract

Adamantinomatous craniopharyngioma (ACP) is an epithelial tumor that arises when Rathke’s pouch remains during embryonic development. The pathogenesis of ACP remains unclear, and treatment options are limited. Here, we reveal the critical genes expressed in ACP and provide a basis for further research and treatment. The raw dataset GSE94349 was downloaded from the GEO database. We selected 24 ACP and 27 matched samples from individuals with no documented tumor complications (control group). Then, we screened for differentially expressed genes (DEGs) to identify key signaling pathways and associated DEGs. A total of 470 DEGs were identified (251 upregulated and 219 downregulated). Hierarchical clustering showed that the DEGs could precisely distinguish the ACP group from the control group (CG). Gene Ontology (GO) enrichment analysis indicated that the upregulated DEGs were mainly involved in cell adhesion, inflammatory responses, and extracellular matrix management. The downregulated DEGs were primarily involved in cell junction and nervous system development. Kyoto Encyclopedia of Genes and Genomes (KEGG) pathway enrichment analysis indicated that the critical pathway was pathways in cancer. In the PPI network, *CDH1*, *SHH*, and *WNT5A* had the highest degrees of interaction and were associated with the formation of ACP. *CDH1* was verified as a critical gene by quantitative reverse transcription–polymerase chain reaction (qRT-PCR) in ACP and CG samples. We found that *CDH1* may play an important role in the pathways in cancer signaling pathway that regulates ACP development. The *CDH1* gene may be a target for future research and treatment of ACP.

## Introduction

Craniopharyngioma (CP) is a complex and diverse congenital intracranial tumor, and surgical resection is the primary treatment strategy at present. Because this tumor is located near essential brain structures, such as the optic nerve and hypothalamus, it poses significant challenges for surgery. Damage to the hypothalamus and other important brain tissue structures can lead to high fever, electrolyte disorder, obesity, and other severe complications and seriously harm patients’ quality of life after surgery (1). Treatment options for CPs are difficult to determine because the balance of risks and benefits of surgery varies significantly from patient to patient. The close, heterogeneous relationship to the hypothalamus makes surgical removal of CPs challenging even though this remains the primary treatment strategy (1, 2).

The incidence of CPs is 4.6% of all intracranial tumors, and its exact pathogenesis remains unclear (1). There are two theories regarding the origin of CP cells: embryogenetic theory and metaplastic theory (3). There are two histological types of CPs, ACP and papillary craniopharyngioma (PCP), of which ACP is the most common (4). As PCP is rare in clinical practice, all subjects in this study had ACP. Exome sequencing studies have demonstrated that PCP and ACP have distinct genetic origins, primarily driven by mutually exclusive alterations; *BRAFV600E* is observed in 95% of PCPs, and *CTNNB1* is observed in 75%–96% of ACPs (5). For PCPs with *BRAFV600E* mutations, targeted therapy with *BRAF* inhibitors combined with MEK inhibitors can reduce tumor volume by 85% to 91% (6, 7). However, no similar clinical studies have been conducted on ACPs. In addition to *CTNNB1*, *CDH1* and *SHH* may play essential roles in the occurrence of ACP (8, 9).

Gene expression microarrays can be used to identify differentially expressed genes (DEGs) in ACP tissues, providing high-throughput gene expression data. Gene expression microarrays are an essential approach for studying the pathogenesis of CP. The National Center of Biotechnology Information GEO database comprises a large amount of genetic data, providing a rich resource for the study and analysis of differential gene expression levels (10).

Zou et al. (11) analyzed CP data in the USA, revealing that the mechanisms of ACP occurrence and development might involve the regulation of the RNA polymerase II promoter and glutamate receptor binding. Li et al. (12) also analyzed sample data and identified MMP12 as a potential therapeutic target in ACP. The present study examined the GSE94349 dataset, comprising a large number of samples obtained from American patients (13).

GSE94349, submitted by Donson et al. on 31 January 2017 (13), contains genetic data from 168 samples, analyzed by microarray technology using the Affymetrix Human Genome U133 Plus 2.0 array GPL570 platform.

We selected 24 ACP surgical tumor samples and 27 samples from individuals with no documented tumor complications (control group, CG) from GSE94349. However, as Donson et al. (13) was a clinical trial, the researchers did not quickly achieve a perfect match between the two groups. Although these two studies have implications for ACP, our study further contributed GSEA data. Finally, we used the intersection of the Database for Annotation, Visualization, and Integrated Discovery (DAVID) and GSEA to determine the key genes. No relevant article has been published regarding the critical genes involved in ACP by using the intersection of DAVID and GSEA.

By analyzing the gene expression data of GSE94349, DAVID and GSEA were used to conduct GO and KEGG pathway enrichment analyses. Then, the intersection of the two methods was obtained, namely, crucial pathways and genes. Finally, STRING and Cytoscape software were used to construct protein−protein interaction (PPI) networks of critical pathways and genes. The present study identified critical genes in the pathogenesis of CP, which will provide new insight into the treatment of CP.

## Materials and methods

### Gene annotation and missing value supplementation

The gene expression microarray data of GSE94349_series_matrix.txt were downloaded from the GEO (http://www.ncbi.nlm.nih.gov/geo/) database. The quality control and standardization of these data were completed prior to the current study. The data were processed using R software (version3.6; http://www.r-project.org/). First, we searched and downloaded GPL570, the corresponding platform of GSE94349, from the official GEO website. GSE94349 was matched with GPL570 in R language to complete gene ID conversion and annotation. Next, the missing values were calculated by KNN in R language to supplement the missing values.

### Identification of the DEGs

DEGs were filtered by using the limma package in R (http://limma.org) (14). Only genes with | log2-fold change (Fc) | > 4 and *p*-values < 0.01 were considered DEGs. Finally, we obtained upregulated and downregulated DEGs. Volcano mapping was performed in R language; heatmaps were drawn using the R package gplot 2.

### GO enrichment analysis of DEGs

The upregulated and downregulated DEGs were input into the online tool DAVID (DAVID 6.8 version) for GO enrichment analysis. The results were analyzed according to biological process (BP), cellular component (CC), and molecular function (MF), and *p* < 0.01 was considered statistically significant.

### KEGG pathway enrichment analysis

DEGs were imported into the DAVID online tool for KEGG pathway enrichment analysis, and *p* < 0.01 was considered statistically significant. We imported all the gene data for the GSEA. The Kolmogorov−Smirnov test was used to calculate the value of DEGs in each KEGG pathway by 1,000 repeated permutation tests to conduct the KEGG pathway enrichment analysis of DEGs in GSEA. *p* < 0.01 was considered statistically significant. Then, the upregulated and downregulated DEG KEGG pathway enrichment analysis results were derived. Finally, the intersection of the results of the two analysis methods was obtained to determine the target DEGs.

DAVID was used to conduct KEGG pathway enrichment analysis for all the different genes, yielding more comprehensive results. However, GSEA is a KEGG pathway enrichment analysis of all genes, yielding a complete dataset. The intersection of the two may be more valuable for further research (15). We used Venn diagrams to show the common KEGG pathways after the intersection of the DAVID and GSEA results.

### PPI network

The KEGG pathway-related DEGs from the intersection of the DAVID and GSEA results were imported into the Search Tool for the Retrieval of Interacting Genes (STRING). The gene interaction relationship was derived with a confidence score >0.7 as the cutoff standard. Then, Cytoscape software (Version 3.5.1) was used to construct the PPI network between the KEGG pathway and its related DEGs. CytoHubba was used to predict the top 10 key genes. Three critical genes (*CDH1*, *WNT5A*, and *SHH*) were selected.

### Patients and sample collection

ACP and CG samples were provided by the Department of Neurosurgery at The First Affiliated Hospital of Nanchang University (Nanchang, China). ACP samples were collected after endoscopic nasal resection, stored at 4°C for transport and then preserved in liquid nitrogen. Samples from individuals with no documented tumor complications [control group (CG)] were collected from patients undergoing surgery for epilepsy. The mean age of the four epileptic patients was 20 years (age range, 8–34 years), including two men and two women. A total of four ACP samples from two men and two women were obtained. The mean age of the ACP patients was 25.5 years (age range, 8–35 years). Patients pathologically diagnosed with ACP were included in the present study, while patients with other diagnoses were excluded. All specimens were pathologically and clinically diagnosed as ACP by three pathologists. The present study was approved by the Research Ethics Committee of Nanchang University [Nanchang, China; First Affiliated Hospital of Nanchang University (2020) Medical Research Ethics Review (No. 160)], and written informed consent was provided by all patients prior to the start of the study.

### Sample inclusion criteria

#### ACP samples

1. Clinical diagnosis of craniopharyngioma and consent to surgery;
2. Han nationality, Chinese, right-handed;
3. No history of chronic cardiovascular diseases; no history of infectious diseases; previously healthy, no history of kidney disease and liver disease;
4. Normal hearing and language functions, no metal implants in the body, no history of surgery for heart, lung, or other vital organs, no history of major diseases such as brain disorders, and no history of mental illness; and
5. At least two pathologists diagnosed ACP.
6. Only when the above five items meet the requirements can they be included in the sample database of this study.

#### CG samples

1. Clinically diagnosed epilepsy and consent to surgery;
2. Han nationality, Chinese, right-handed;
3. No history of chronic cardiovascular diseases or infectious diseases;
4. No history of kidney and liver diseases;
5. Normal hearing, no metal implants in the body; and
6. No history of mental illness.
Only when the above six items meet the requirements can they be included in the sample database of this study.

#### Sample exclusion criteria

1. Oral hormone medication within 2 weeks;
2. Speech and hearing impairment;
3. Patients who cannot cooperate with researchers in information collection (e.g., coma patients with severe illness);
4. Lost contact or refused to answer the phone during the follow-up period so that postoperative information could not be collected; and
5. Patients with anemia, cachexia, and other blood cells or low hemoglobin.
6. Any one of the above five items should be excluded from this study.

#### Exit criteria

1. Patients or their family members voluntarily withdraw from the study after surgery;
2. Non-craniopharyngioma patients were diagnosed after preoperative collection of craniopharyngioma patient specimens;
3. Postoperative complications that require hormone and other related treatments;
4. Postoperative complications and inability to cooperate with follow-up investigators; and
5. Patients who cannot cooperate with postoperative follow-up.
6. If any of the above five criteria are met, the study will automatically stop by default.

### RT-PCR

Three critical genes (*CDH1*, *WNT5A*, and *SHH*) were validated in ACP tissues and compared with CG tissues by qRT-PCR using the QuantStudio 7 Flex real-time PCR system (Bio-Rad, Nanchang, China). The primer sequences used are shown in **Table 1**. The 2^−ΔΔCt^ method was used, and the PCR results were normalized to the ACTIN gene (16).

**Table 1:** Primers used in present study.

Gene	Forward primer	Reverse primer
SHH	AGGAGTGAAACTGCGGGTGA	CACCGAGCAGTGGATATGTGC
CDH1	ATTGCTCACATTTCCCAACTCC	CTCTGTCACCTTCAGCCATCCT
WNT5A	TGCAATGTCTTCCAAGTTCTTCCT	ATTCATACCTAGCGACCACCAAG
ACTIN	CACCCAGCACAATGAAGATCAAGAT	CCAGTTTTTAAATCCTGAGTCAAGC

Total RNA was extracted from ACP samples according to the instructions of the RNA Extraction Kit (Servicebio, China). Then, the total RNA of ACP samples was reverse transcribed according to the steps of the Servicebio^®^RT First Strand cDNA Synthesis Kit (Servicebio, China). The total RNA and cDNA of CG samples were obtained by the same method. Finally, 7.5 μl of 2×qPCR Mix, 1.5 μl of 2.5 μm primer (upstream + downstream), 2.0 μl of reverse transcription product (cDNA), and 4.0 μl of ddH_2_O were added to a 200-µl PCR tube. After the PCR solution was prepared, it was gently mixed by pipetting up and down. A 96-well PCR plate was placed into a special bracket. The researchers avoided touching the bottom of the reaction plate to avoid affecting the data reading. Three wells were prepared for each reaction, and qPCR was conducted using the following reaction conditions: predenaturation at 95°C for 10 min, denaturation at 94°C for 15 s, and annealing at 60°C for 30 s for 40 cycles. When the temperature rose from 65°C to 95°C, the fluorescence signal was collected every 0.5°C to form a melting curve. RT-PCR (Bio-Rad, China) was performed on CG samples and ACP samples according to the above steps. Each experiment was repeated three times.

### Data analysis

The ΔΔCT method was used as follows: A = CT (target gene, sample to be tested) − CT (internal marker gene, sample to be tested); B = CT (target gene, control sample) − CT (internal standard gene, control sample); K = A − B; express multiple = 2^−K^. All experimental data are expressed as the mean ± SD and were statistically analyzed by *t*-test. *p* < 0.05 was considered to be statistically significant.

## Results

### Gene annotation and missing value supplement

The gene expression microarray data of GSE94349_series_matrix.txt were downloaded from GEO, comprising 168 samples. The ACP and control groups were selected from GSE94349_series_matrix.txt, and other samples were removed. Quality control and standardization of these data were completed, and all chip data reached comparable levels.

### Identification of DEGs

**Figure 1:**
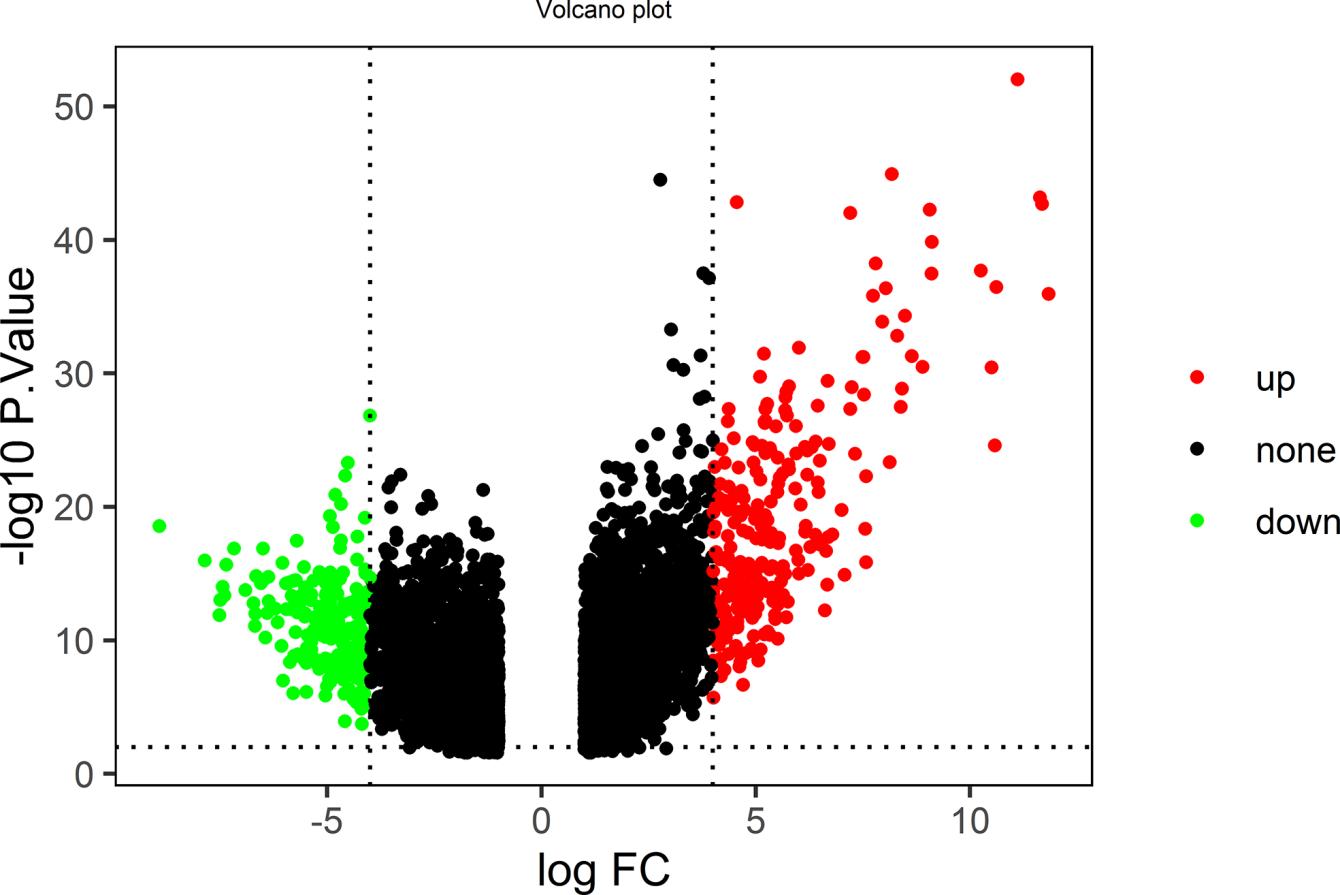
The volcano plot shows all genetic differences with a threshold of −log10 *p* > 2 (*p* < 0.01) and |log2 Fc| > 4; red dots represent upregulated genes, and green dots represent downregulated genes.

Twenty-four ACP samples and 27 control samples in the GSE94349 dataset were analyzed. The volcano plot in **Figure 1** shows all genetic differences with a threshold of -log_10_ *p* > 2 (*p* < 0.01) and |log_2_ Fc| > 4. Red dots represent upregulated genes, and green dots represent downregulated genes. Then, based on the cutoff criteria (*p* < 0.01 and |log2 Fc| > 4), a total of 470 DEGs were identified, including 251 upregulated and 219 downregulated DEGs.

**Figure 2:**
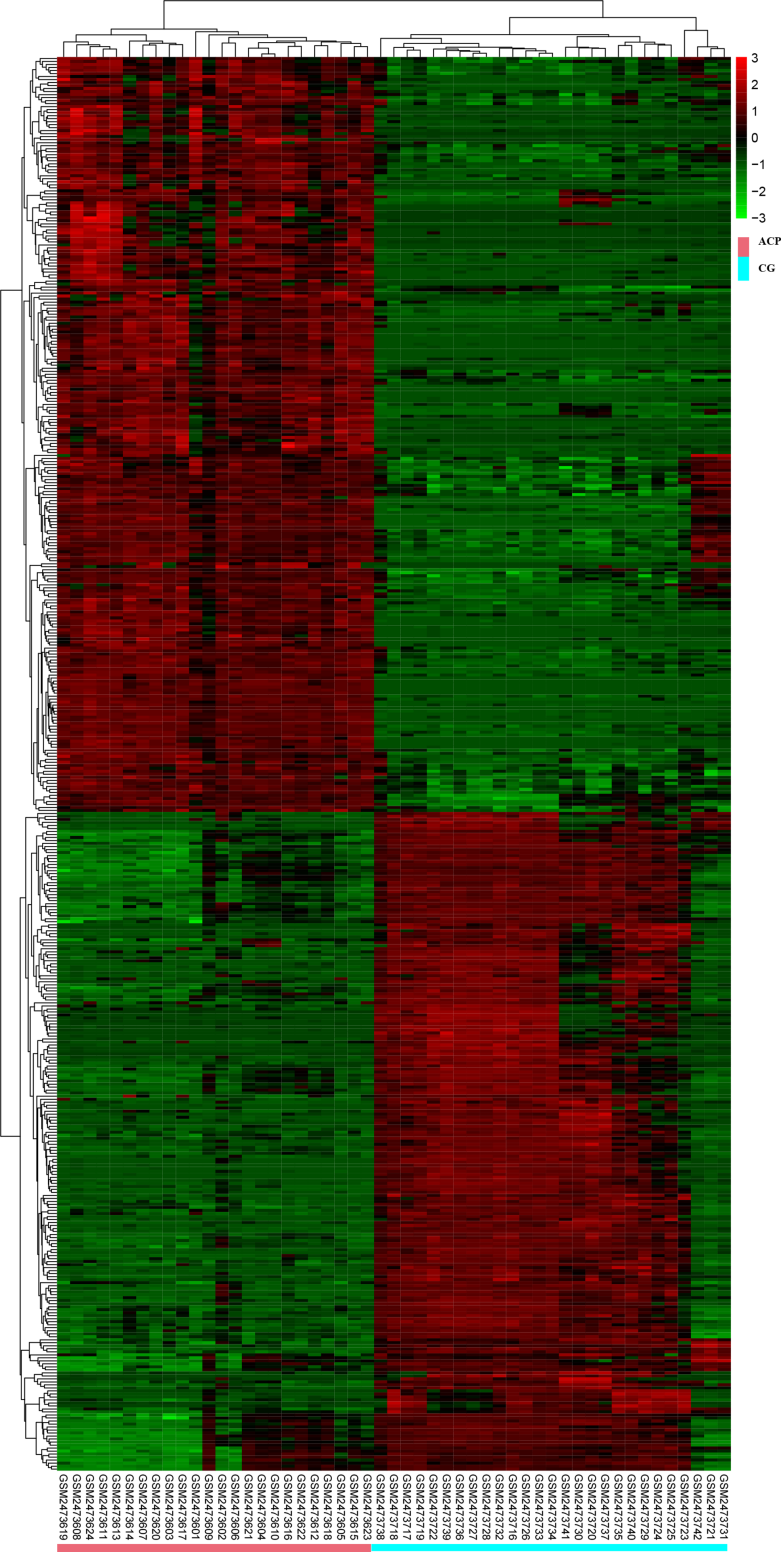
Hierarchical cluster analysis could accurately distinguish ACP samples from control samples. CGs, individuals with no documented tumor complication samples; ACP, adamantinomatous craniopharyngioma.

All DEGs were analyzed. DEGs were also divided into upregulated and downregulated components for separate analyses. This study aimed to identify the upregulation and downregulation of target DEGs; thus, we divided the upregulated and downregulated genes for analysis. The expression heatmap of the DEGs is shown in **Figure 2**. Hierarchical cluster analysis accurately distinguished ACP samples from control samples.

### GO enrichment analysis

**Figure 3:**
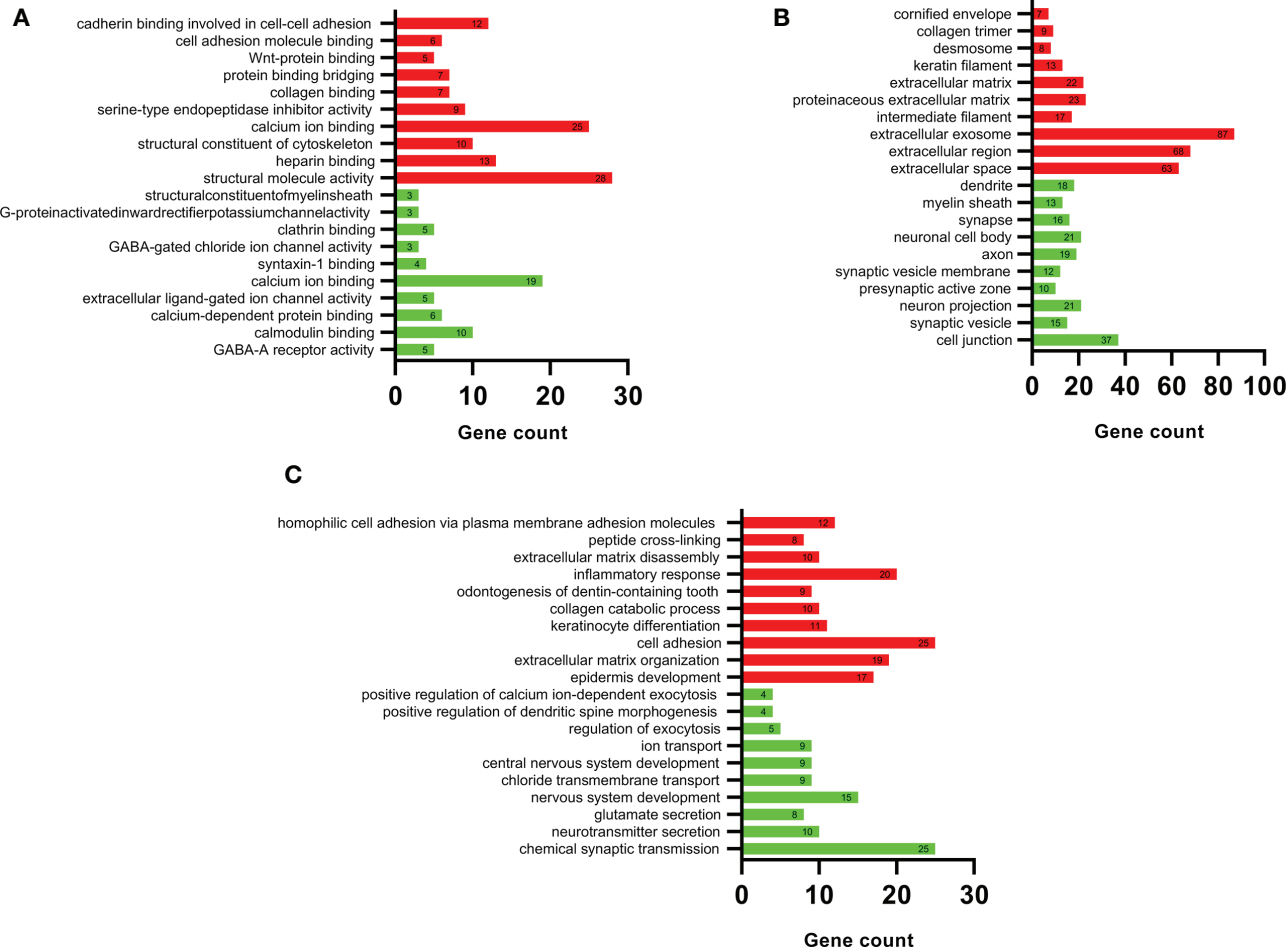
DAVID GO enrichment analysis results. According to the three functions of **(A)** molecular function, **(B)** cellular component, and **(C)** biological process, the top 10 upregulated (red) and top 10 downregulated (green) terms in each category.

The DEGs were imported, DAVID was used for functional enrichment analysis, and the results were obtained according to the three functions of MF, CC, and BP. The GO analysis results of upregulated and downregulated DEGs are listed in the top 10 items in order of *p*-value in **Figure 3**.

**Figure 3A** shows the detailed results of the MF analysis of DEGs. The upregulated DEGs were mainly related to the activation of structural molecules and the binding of calcium ions; downregulated DEGs were mainly associated with calcium ions and calmodulin binding. **Figure 3B** shows the detailed results of the CC analysis of DEGs. The upregulated DEGs were mainly concentrated in exosomes, extracellular regions, and extracellular spaces; downregulated DEGs were mainly enriched in cell junctions, neuron projections, and neuronal cell bodies.

**Figure 3C** shows the detailed results of the BP analysis of DEGs. The upregulated DEGs were mainly associated with cell adhesion, inflammatory responses, and extracellular matrix organization, and the downregulated DEGs were mainly associated with chemical synaptic transmission and nervous system development.

The results showed that the upregulated genes were mainly involved in cell adhesion, inflammatory responses, and extracellular matrix management. The downregulated genes were mainly involved in cell junction and nervous system development.

### Pathway enrichment analysis

**Figure 4:**
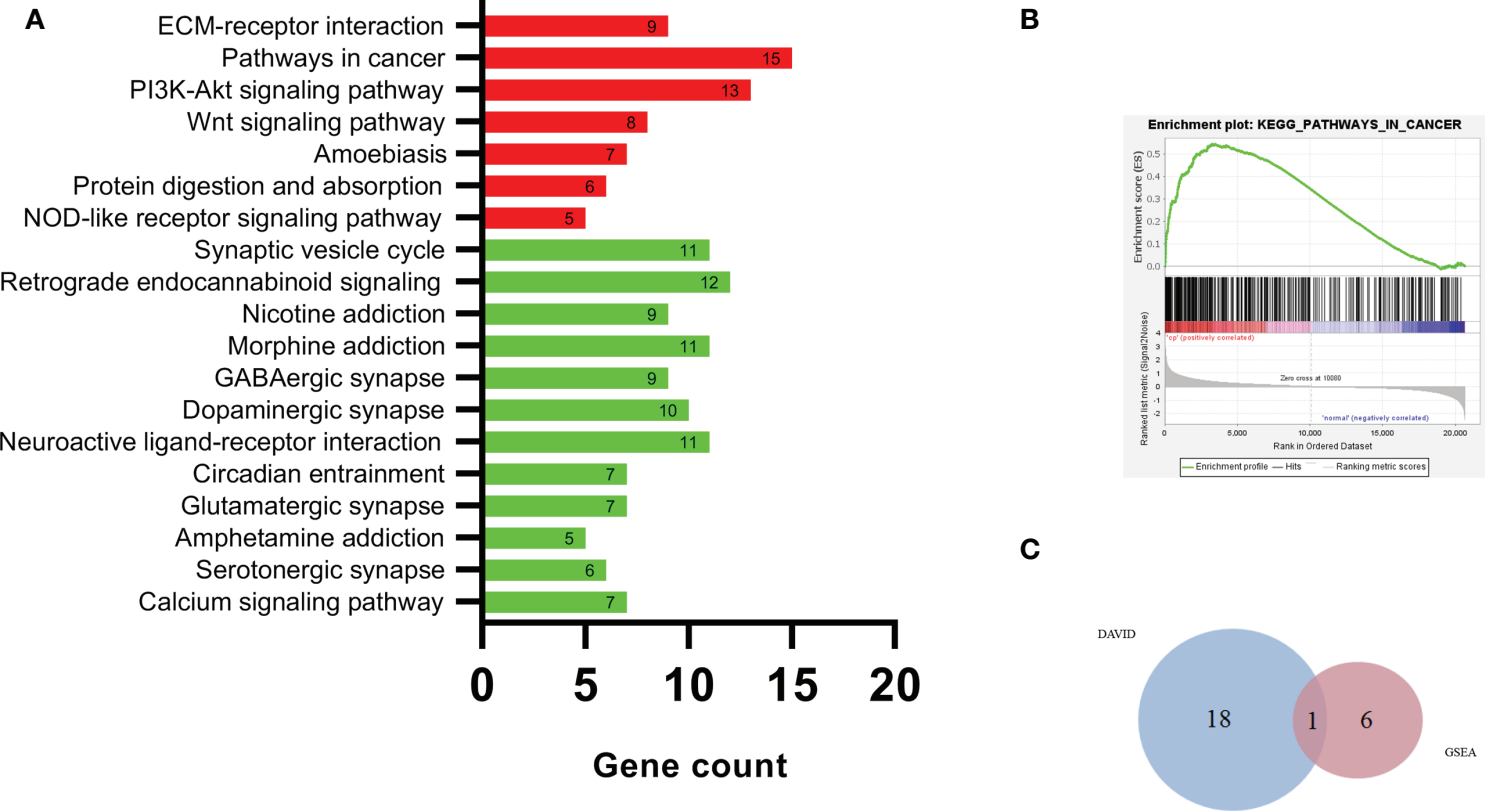
The DEG KEGG pathway analysis by DAVID **(A)**, pathways in cancer **(B)**, and Venn plot of DAVID and GSEA **(C)**. KEGG, Kyoto Encyclopedia of Genes and Genomes; DAVID, Database for Annotation Visualization and Integrated Discovery; GSEA, Gene Set Enrichment Analysis.

**Figure 4A** shows the results of the DEG KEGG pathway analysis by DAVID. Seven KEGG pathways were enriched in the upregulated DEGs (*p* < 0.01). Twelve KEGG pathways were enriched among the downregulated DEGs (*p* < 0.01).

The upregulated pathway comprised pathways in cancer, the PI3K-Akt signaling pathway, the extracellular matrix (ECM)–receptor interaction, and the Wnt signaling pathway. The downregulated pathways were mainly associated with pathways related to retrograde endocannabinoid signaling, the synaptic vesicle cycle, morphine addiction, and neuroactive ligand–receptor interactions.

**Figure 4B** and **Table 2** show the results of DEG analyses obtained by GSEA KEGG pathway analysis. Using *p* < 0.01 as the standard, there were seven upregulated and no downregulated pathways. Detailed results of the 7 KEGG pathways are shown in **Table 2**.

**Table 2:** KEGG enrichment analysis results (GSEA).

Pathway name	Gene number of pathway	ES	NES	NOM *p*-value	FDR *q*-value
Small cell lung cancer	82	0.652	1.628	0.000	0.605
Pathways in cancer	311	0.545	1.604	0.000	0.516
P53 signaling pathway	63	0.675	1.579	0.002	0.328
Steroid hormone biosynthesis	45	0.506	1.550	0.004	0.234
Cytokine–cytokine receptor interaction	240	0.656	1.548	0.004	0.200
Tgf beta signaling pathway	82	0.568	1.523	0.008	0.217
Riboflavin metabolism	15	0.621	1.572	0.009	0.299

KEGG, Kyoto Encyclopedia of Genes and Genomes; GSEA, gene set enrichment analysis; ES, enrichment score; NES, normalized enrichment score; NOM, nominal; FDR, false discovery rate.

Upregulated pathways were mainly associated with pathways in cancer and cytokine−cytokine receptor interactions.

**Figure 5:**
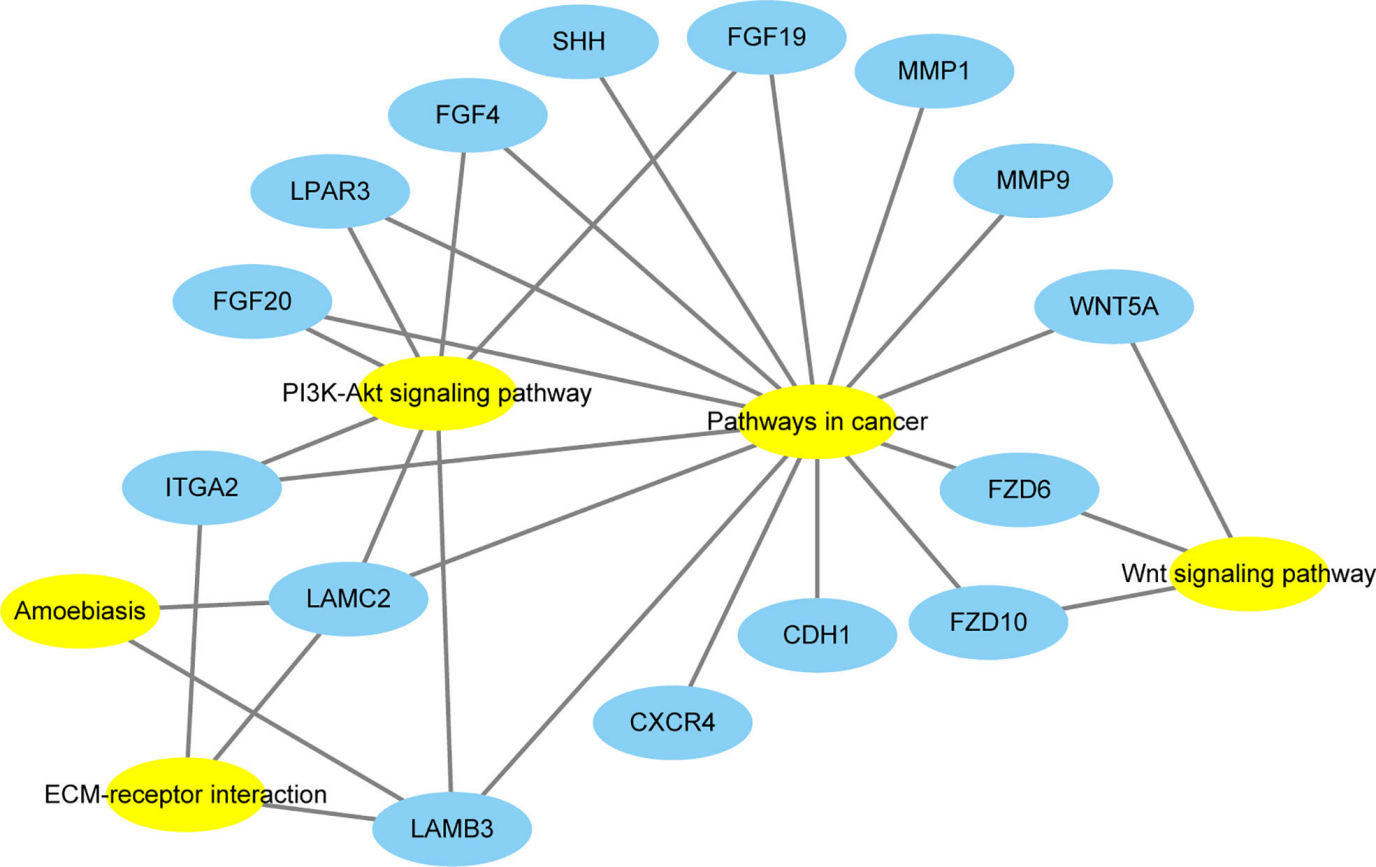
The relationship between this unique pathway (Pathway in cancer) and related pathways and genes. Yellow represents the pathways, and blue represents the genes.

The KEGG pathway analyses identified 19 significant pathways from DAVID and seven significant pathways from GSEA. The results of the two methods intersected, and only one common upregulated pathway was found, as shown in the Venn diagram in **Figure 4C**. The only upregulated pathway was “pathways in cancer”. The relationship between this unique pathway and related pathways and genes is shown in **Figure 5**.

### PPI network analysis

**Figure 6:**
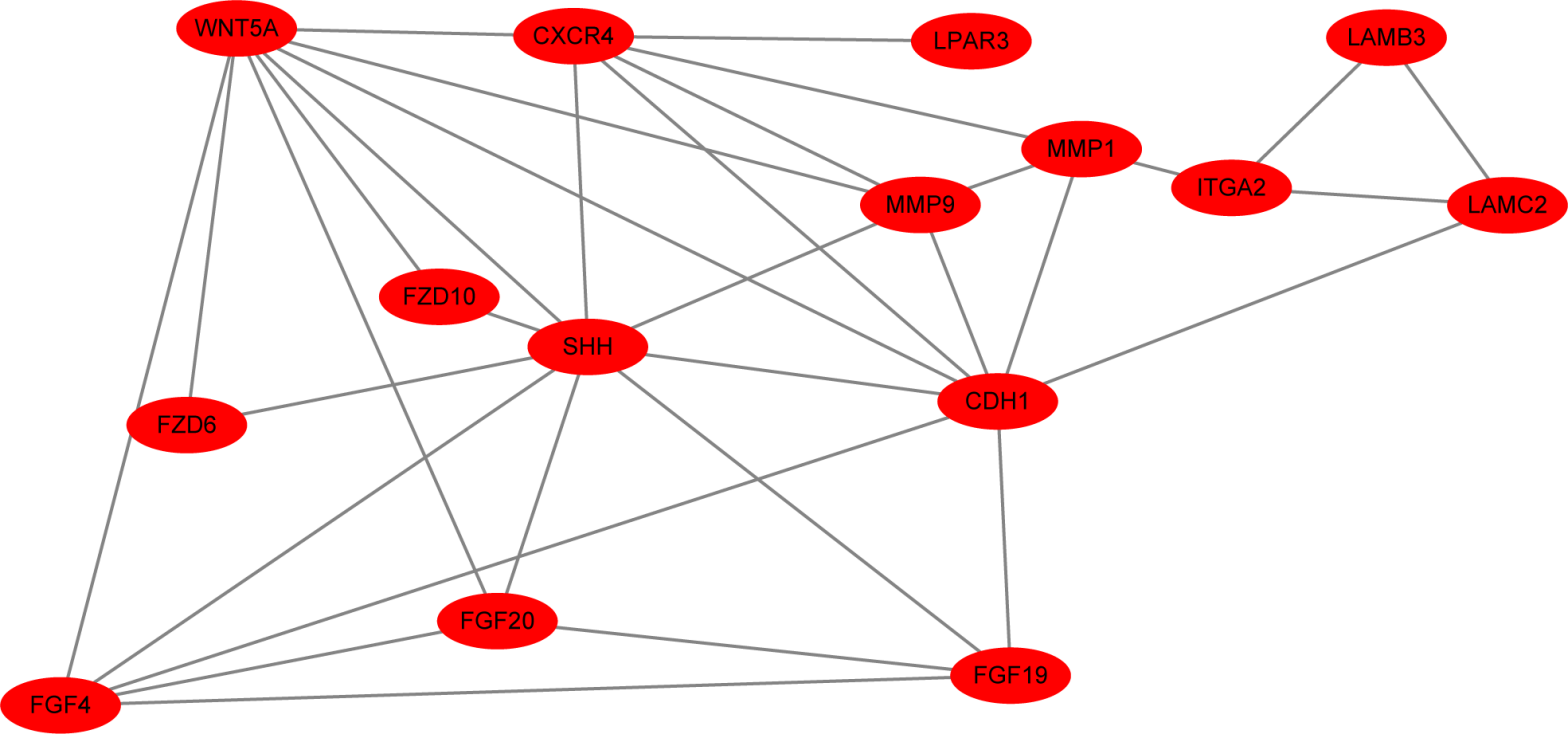
The interaction score was >0.7, and the PPI network of all upregulated DEGs (15 nodes and 33 edges) is shown.

**Figure 7:**
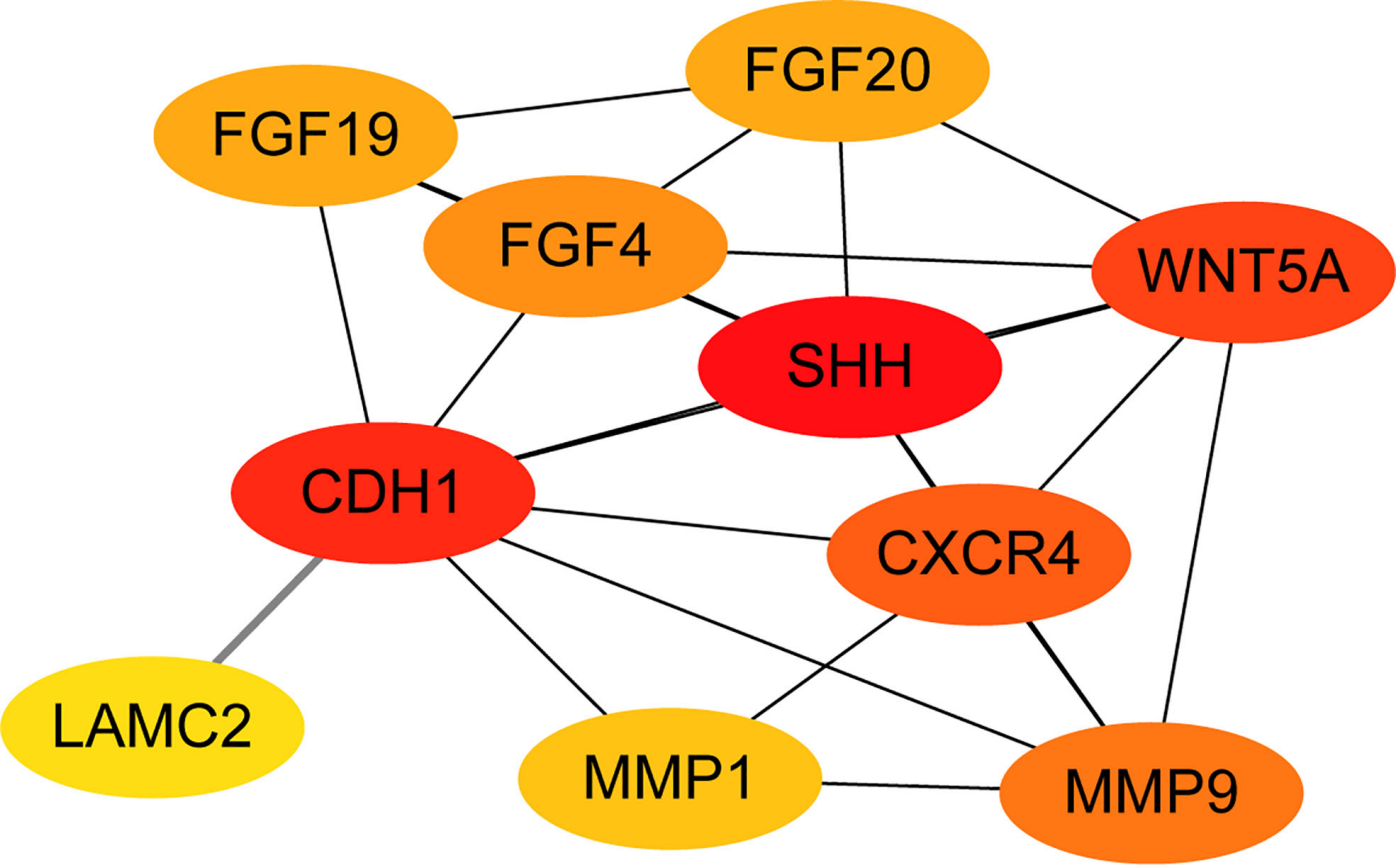
The PPI network diagram was constructed from the top 10 key genes among the 15 DEGs.

All of the 15 DEGs in the one common pathway were collected using STRING, and a PPI analysis was performed. The 15 DEGs were imported into the STRING database, and their interactions were identified. If the interaction score was >0.7, the PPI network could be formed with 14 nodes and 32 edges. The PPI networks presented in **Figure 6** show that all DEGs were upregulated. As shown in **Figure 7**, the cytoHubba plug-in was used in Cytoscape software to search for the top 10 key genes among the 15 DEGs in the PPI network map. Finally, the top three critical genes (*CDH1*, *WNT5A*, and *SHH*) were selected from the 10 genes for RT-PCR verification.

### RT-PCR results

**Figure 8:**
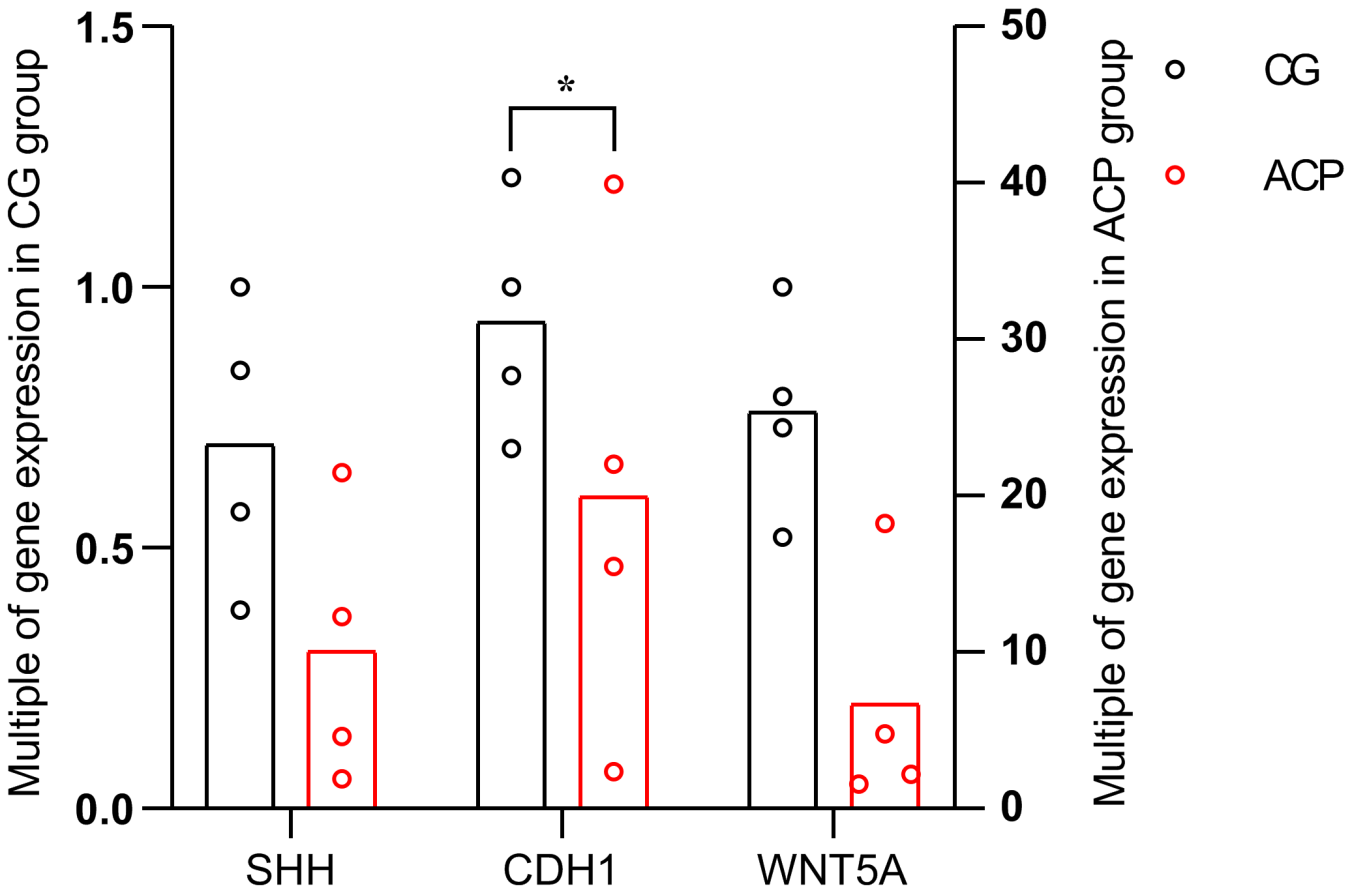
The horizontal axis shows the three key genes in the ACP and control groups. The vertical axis shows the mean value of 2^−ΔΔCT^ of the three key genes in the ACP and control groups. CGs, individuals with no documented tumor complication samples; ACPs, adamantinomatous craniopharyngioma samples; *p < 0.05.

Quantitative reverse-transcription polymerase chain reaction (qRT-PCR) was used to measure and compare the expression levels of the key genes in the ACP and control groups (**Figure 8**). The expression levels of three key genes were significantly increased in ACP *vs*. the control group, including *CDH1*, *WNT5A*, and *SHH*, but only CDH1 had statistical significance (*p* < 0.05) (**Figure 8**).

## Discussion

A total of 470 DEGs were identified in the present study, including 251 upregulated and 219 downregulated DEGs. GO enrichment analysis indicated that the upregulated genes were mainly located in the extracellular exosome, extracellular region, and extracellular space and were involved in structural molecule activation and the binding of calcium ions, cell adhesion, inflammatory responses, and extracellular matrix organization bioprocesses. Another study showed that proinflammatory mediators drive the phosphorylation and activation of STAT3 (17). Persistent signaling through this pathway in cancer can result in a chronic inflammatory phenotype and suppression of antitumor immunity (13). Other studies have shown that cholesterol crystals in ACP activate the inflammasome, leading to the secretion of inflammatory cytokines that drive the inflammatory response (18). In addition, ECM proteins mediate epithelial–mesenchymal transformation (EMT) in ACP cells (19). The downregulated genes were mainly involved in cell junction and nervous system development. Research has shown that epithelial cell adhesion molecule (Ep-CAM) expression in craniopharyngioma could be a predictive marker of relapse (20). Additionally, ACP cells originate from remnants of Rathke’s cleft, which is neuroepithelial (1, 21).

The KEGG pathway enrichment analysis in this study showed that “pathways in cancer” (https://www.kegg.jp/pathway/hsa05200) was the major pathway involved in ACP.

There was only one common pathway and all 15 DEGs in this pathway were collected using STRING, and a PPI analysis was performed. In the DAVID KEGG analysis, these 15 DEGs were mainly involved in the following five signaling pathways: Wnt signaling pathway, ECM–receptor interaction, amoebiasis, PI3K-Akt signaling pathway, and pathways in cancer. Among these signaling pathways, the association between amoebiasis and ACP was not previously reported. The other four signaling pathways have been associated with ACP.

The Wnt signaling pathway is an important aspect of ACP pathogenesis. *CTNNB1*-Mut has been shown to promote ACP primary cell proliferation by activating Wnt/β-catenin signaling (22). On the other hand, reducing the expression of β-catenin can significantly inhibit the proliferation of ACP cells, and β-catenin can promote the expression of Fascin mRNA and Fascin by acting on Fascin genes in the nucleus, thus promoting the migration and invasion ability of ACP cells (23).

The ECM can mediate EMT in ACP cells (19). EMT plays an important role in ACP development. ECM can facilitate the migration and differentiation of cells and trigger EMT, which is important for the progression and metastasis of various tumors (24, 25). Overexpression of ECM in ACP cells can promote tumor proliferation, migration, and invasion (19). Although the association between the PI3K-Akt signaling pathway and ACP has not been reported, Andoniadou et al. showed that fibroblast growth factors (FGFs) could induce β-catenin cells to actively divide (26). As shown in **Figure 5**, three FGF members (*FGF4, FGF19*, and *FGF20*) of the 15 DEGs were involved in the PI3K-Akt signaling pathway. The FGF family proteins are key regulators of several biological processes, and together with their receptors, they affect the development of many human cancers (27). Overactivation mutations in FGF receptors have been identified in several human cancers, including breast, bladder, and prostate cancers (28, 29). It is tempting to speculate that FGFs induce active division of β-catenin cells through the PI3K-Akt signaling pathway. However, three FGF members of the 15 DEGs were also involved in pathways in cancer; therefore, FGFs induce active division of β-catenin cells through the PI3K-Akt signaling pathway or pathways in cancer, which requires confirmation in further experiments.

Similarly, the association between pathways in cancer and ACPs has not been reported, but the three most significant key genes (*CDH1*, *WNT5A*, and *SHH*) have been closely/positively associated with craniopharyngioma. One report showed that *SHH* is highly expressed in ACPs (9). After the formation of Rathke’s sac, the expression of SHH in this region gradually decreases (30). SHH can promote β-catenin overexpression in ACP animal and cell models by paracrine and autocrine effects, respectively (26). In addition, studies have shown that the expression of *VEGF* in ACP cells can promote tumor angiogenesis and increase microvascular density (31); however, whether the *SHH* pathway affects tumor blood supply in ACP through *VEGF* has not been clarified. This result provides new directions for future research on ACPs.

As a sequestering protein of β-catenin, E-cadherin (*CDH1*) plays an important role in canonical Wnt signaling (32). Previous studies have reported conflicting results regarding the expression of E-cadherin, encoded by the *CDH1* gene, in ACP. Preda et al. found that *CDH1* expression in ACPs was not correlated with β-catenin (33). However, Qi et al. found that β-catenin was positively correlated with *CDH1* expression in ACP; β-catenin might regulate *CDH1* expression in ACPs, and decreased *CDH1* expression in ACPs has been associated with tumor recurrence (34). However, Barros et al. found that *CDH1* expression in ACPs was not associated with tumor recurrence (32). All these findings need to be further verified. However, our results provide a new direction for the future treatment of ACP.

Cancer-associated fibroblasts (CAFs) are a major component of the cancer stroma and promote cancer cell aggressiveness by secreting multiple factors (35). *WNT5A* was found to be highly expressed in tumor stromal fibroblast gastric cancer studies and was associated with poor prognosis (36). Wnt/β-Catenin and *WNT5A*/Ca pathways regulate proliferation and apoptosis of keratinocytes in psoriasis lesions (37). It is tempting to speculate that the Wnt/β-Catenin and *WNT5A*/Ca pathways regulate the proliferation and apoptosis of cells, representing a potential new therapeutic target for treating ACP in the future. However, no similar study has been performed for ACP. Although the key genes revealed by RT-PCR in this study are relatively clear, the sample size was small. A larger number of samples are needed for validation. The roles of the three key genes remain unclear and require verification in future studies. WNT5A could be used as a target for the treatment of ACP in the future.

In conclusion, we hope that these methods can be applied in future studies. DAVID was used to thoroughly analyze the DEGs, while GSEA was performed to comprehensively analyze the genes. The main aim of our study was to analyze the KEGG pathways of all genes, including the DEGs, using GSEA. The analysis of large amounts of ACP gene data requires substantial work, and the analysis results are likely to change as the major databases are updated.

Our results suggest that the 15 DEGs, including *CDH1*, *WNT5A*, and *SHH* genes and “pathways in cancer”, may be associated with ACP. According to the cytoHubba statistical results in Cytoscape software, the first three DEGs were selected for RT-PCR verification. Our results suggest that CDH1 may play an important role in the pathways in cancer signaling pathway that regulate ACP development. However, its specific role in ACP remains to be confirmed in further experiments. Brastianos et al. showed that in PCPs with the *BRAFV600E* mutation, a *BRAF* inhibitor combined with MEK inhibitor targeted therapy can reduce tumor volume by 85%–91% (6, 7). We expect that ACP can be eliminated by similar treatments in the future. The limitation of this study is that most of the conclusions were drawn from bioinformatics analyses, and previous experimental results are lacking in in-depth research.

Clinical craniopharyngioma is rare. Our neurosurgery department treats approximately 12 patients with craniopharyngioma annually, among which 2–3 have PCP and 1–2 decline surgical treatment. Therefore, it is difficult to obtain a large number of samples for validation in a short time period. In addition, at present, methods for craniopharyngioma cell culture are in the early stages; thus, the characteristics of these tumors are challenging to verify in cell experiments.

In addition, no other analytical methods were used in this study; thus, more reliable conclusions may be obtained by integrating other analytical methods, such as ingenuity pathway analysis and WebGestalt analysis. For the three key genes selected in this study, we hope to further verify these three critical genes identified in this study and explore their specific mechanisms of action in ACP. In particular, we expect that CDH1 will be studied in the future to determine how it regulates the occurrence and development of ACP through the pathways in cancer signaling pathway.

According to the results of this study, we concluded that pathways in cancer signaling pathway is an important signaling pathway in the development of ACP. CDH1, WNT5A, and SHH regulate ACP formation through this pathway. Although this study concludes that CDH1 is the most critical gene, due to the small sample size of this study, we will expand the sample size in the future to further verify this result. For the study of ACP, in addition to CTNNB1 mutation expressing β-catenin to activate the classical Wnt signaling pathway and promote the proliferation and migration of tumor cells, current studies have confirmed that SHH in ACP can also participate in the regulation of the Wnt signaling pathway by promoting the expression of β-catenin through autocrine or paracrine. At the same time, β-catenin can regulate the expression of EGFR and promote the migration of tumor cells (38–40). Unfortunately, these studies have only been carried out in animals and cells and have not been confirmed in clinical trials. In addition, due to the lack of stable ACP cell lines, although many researchers have extracted primary ACP cells from fresh ACP tissues, no stable ACP cell lines have been constructed, so the results of these cell experiments are difficult to accept by the public.

## Data availability statement

Publicly available datasets were analyzed in this study. This data can be found here: https://www.ncbi.nlm.nih.gov/geo/query/acc.cgi?acc=GSE94349.

## Ethics statement

This study was reviewed and approved by The Research Ethics Committee of Nanchang University [NanChang, China; First Affiliated Hospital of Nanchang University (2020) Medical Research Ethics Review (No. 160)]. Written informed consent to participate in this study was provided by the participants’ legal guardian/next of kin.

## Author contributions

TH and SZ conceived the study and supervised the research. CF performed the bioinformatics analysis and was a major contributor to the writing of the manuscript. LZ prepared the figures and edited the manuscript. HH performed the PCR validation experiments. All authors contributed to the article and approved the submitted version.

## Funding

This study was supported by the National Natural Science Foundation of China (grant no. 82060246), the Natural Science Foundation of Jiangxi Province, and the Science and Technology Research Project of Jiangxi Provincial Education Department (grant nos. 20202BABL206059 and GJJ190128).

## Conflict of interest

The authors declare that the research was conducted in the absence of any commercial or financial relationships that could be construed as a potential conflict of interest.

## Publisher’s note

All claims expressed in this article are solely those of the authors and do not necessarily represent those of their affiliated organizations, or those of the publisher, the editors and the reviewers. Any product that may be evaluated in this article, or claim that may be made by its manufacturer, is not guaranteed or endorsed by the publisher.
